# Fatigue in multiple sclerosis: can we measure it and can we treat it?

**DOI:** 10.1007/s00415-024-12524-9

**Published:** 2024-07-05

**Authors:** John DeLuca

**Affiliations:** 1 Kessler Foundation, 1199 Pleasant Valley Way, West Orange, NJ 07052 USA; 2 Department of Physical Medicine and Rehabilitation, New Jersey Medical School, Rutgers University, Newark, NJ USA

**Keywords:** Multiple sclerosis, Fatigue, Cognitive fatigue

## Abstract

Fatigue is a common and debilitating symptom in multiple sclerosis (MS). However, after over 100 years of inquiry its definition, measurement and understanding remains elusive. This paper describes the challenges clinicians and researchers face when assessing and treating MS patients, as well as our understanding of neural mechanisms involved in fatigue. Challenges for the future are discussed.

## Introduction

Fatigue is the most common symptom in persons with MS, with an incidence of up to 90% of patients and frequently significantly impacts on everyday life activities. Fatigue is a concept we all know but is inconsistent and difficult to define. Its meaning is often vague and difficult to operationalize [4]. After over 100 years of inquiry, its definition remains elusive, and some have suggested the term be abandoned (e.g., Muscio [20], Hubbard [27]). Years ago, Muscio [27] concluded that “it is obviously absurd to set about finding a test of an undefined entity”. Eighty years later Dittner et al. [12] stated: “Before a concept can be measured, it must be defined, and before a definition can be agreed, there must exist an instrument for assessing phenomenology. There is unfortunately no ‘gold standard’ for fatigue, nor is there ever likely to be”. Today, after more than 100 years of inquiry, our ability to define, measure and treat fatigue has improved marginally.

Defining Fatigue

The multidimensional nature of fatigue has been known for over 100 years encompassing: Behavior (i.e., performance), Feeling state (i.e., subjective sense), Mechanism, and Context [26]. Yet, clinical medicine focuses solely on subjective complaints of fatigue. However, even defining and measuring subjective fatigue has been challenging. For instance, in MS, chronic fatigue has been defined as “fatigue that is present for any amount of time on 50% of days for more than 6 weeks, that limits functional activities or quality of life” Multiple Sclerosis Council [16]. However, in chronic fatigue syndrome (CFS) it is defined as “New onset of unexplained, persistent or relapsing fatigue for at least 6 months …” (not result of ongoing exertion, not substantially alleviated by rest, substantial reduction in level of functioning). This marked lack of a clear definition of fatigue is pervasive across clinical entities.

There are dozens of definitions of fatigue, which themselves illustrate the problem (c.f., [10] Supplement 2 for examples). Unfortunately, none truly captures the complexity of the construct. Chaudhuri and Behan [8] offer a definition which at a minimum is testable, defining central fatigue as: “the failure to initiate and/or sustain attentional tasks (‘mental fatigue’) and physical activities (‘physical fatigue’) requiring self-motivation (as opposed to external stimulation)”.

Measuring fatigue

Despite lacking a clear definition, Hjollund et al. [19] found over 250 scales to measure fatigue. Such instruments are used clinically to assess subjective fatigue across various populations. Close et al. [10] analyzed the most frequent fatigue inventories used in MS and found that they lacked content validity, raising the question as to whether they actually assess fatigue? There are numerous problems with such instruments which typically become dogmatically unchallenged. The first is that many assess fatigue without defining it (e.g., FSS). More problematically, fatigue inventories often (if not most of the time) include items that have little or nothing to do with fatigue [10]. For instance, questions regarding sleep are often included despite having distinct neural representations and is distinguishable from fatigue [32]. Questions regarding reduced cognitive functioning are often included despite no clear or consistent evidence that fatigue reduces cognitive functions, although some studies show a relationship with intraindividual variability [30]. The fact is that most studies show that fatigue does not affect objective cognition (e.g., Lohaus et al. [22]), and in some cases even improves cognitive performance (e.g., Chang et al. [7]) including in other populations [2]. In truth, “Under fatiguing conditions, performance sometimes declines, sometimes remains unchanged, or sometimes even increases as time on task increases.” (Ackerman [1], p.3).

Another problem is that current subjective fatigue inventories measure “trait” fatigue, that is self-reported fatigue over a period of time such as the past week (e.g., FSS) or past 4 weeks (e.g., MFIS). However, fatigue in the moment (i.e., “state” fatigue) can be distinguished from “trait” fatigue. The problem here is that state and trait fatigue typically do not correlate (e.g., Sandry et al. [33], Wylie et al. [32]) or correlate poorly [18], even in other populations (e.g., Malloy et al. [23], Moller et al. [24]) begging the question: Is fatigue, fatigue?

Some questions

Why should subjective fatigue be the gold standard for its measurement? Sure, it is easy to use a fatigue inventory, but what use is it if its truly not measuring the desired construct [10]? Why should objective and subjective fatigue correlate? The truth is that clearly the literature shows that they don’t. Why must behavioral performance result in a decline with increasing fatigue? The data supporting this is not strong. This suggests that the concept of “fatigability” which, by definition requires a decrease in performance, may be flawed in that a decrease is often not observed. Perhaps we should return to the traditional term of “performance” which allows for either decreases, no change or increases, rather than fatigability. Let’s face it, subjective fatigue correlates with other subjective ratings such as depression, pain, subjective sleep, cognitive complaints, stress, etc., and is related to other significant factors such as deconditioning, hormonal changes or medication side effects. While the strongest behavioral correlation with fatigue is with depression, it must be recognized that fatigue can be present in the absence of depression. Part of this high correlation is likely due to the overlap between depression and fatigue measures.

Fatigue and the brain

**Fig. 1:**
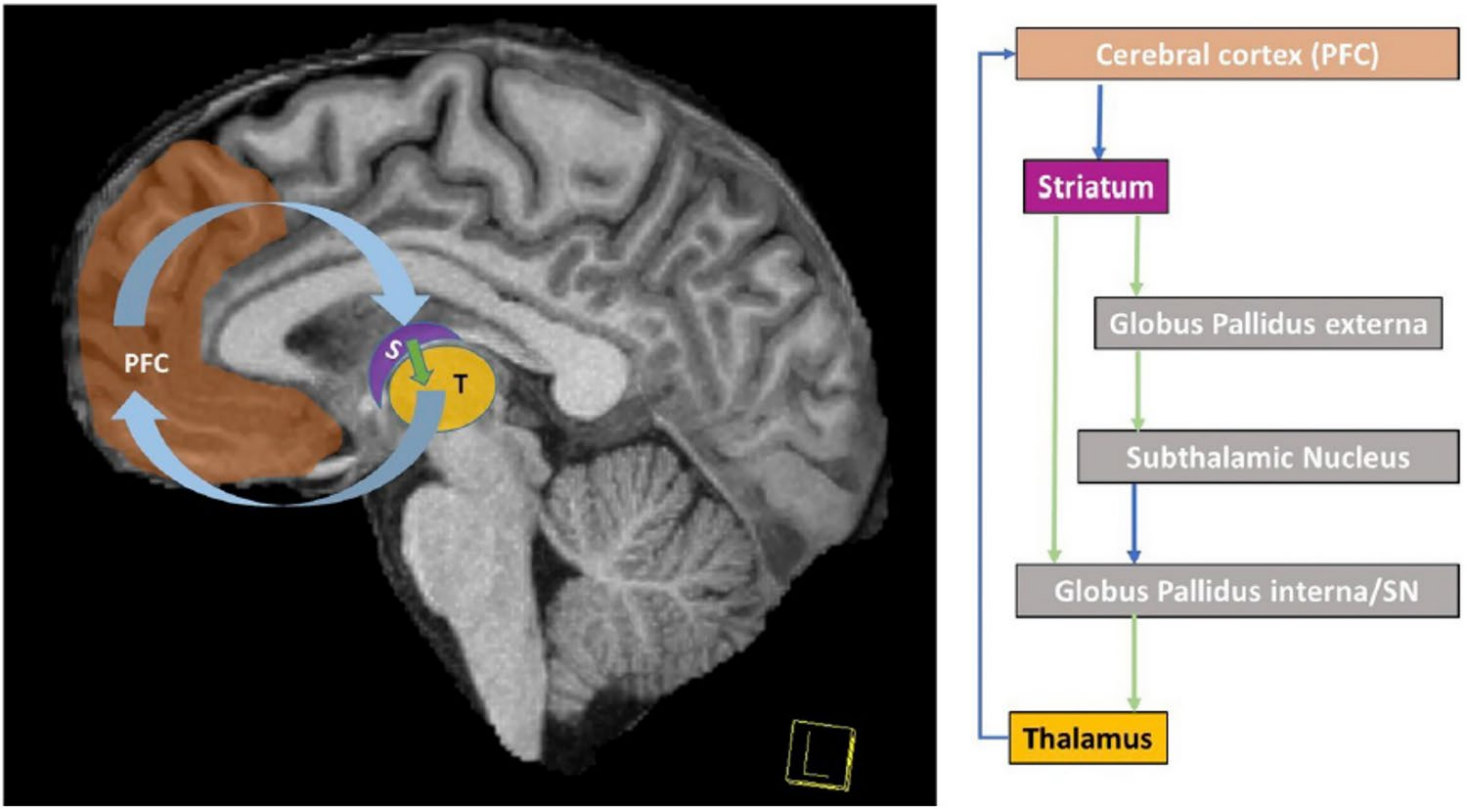
cortico-striato-thalamo-cortical (CSTC) loop underlying fatigue. Need to get permission from Arm et al. [3]

Chaudhuri & Behan [8] hypothesized that central fatigue was due to the “failure in the integration of limbic input and non-motor functions within the basal ganglia affecting the striatal-thalamic-frontal cortical system”. Several studies have supported this hypothesis both in MS (e.g., Arm et al. [3], Román et al. [31]) and in other populations (e.g., Wylie et al. [32]). Advanced MRI imaging techniques have consistently shown that central fatigue, both physical and cognitive, are correlated with disruptions in the cortico-striato-thalamo-cortical (CSTC) loop (see Fig. 1) [3], resulting in a fatigue network [9]. Recent research using signal detection theory shows that persons with MS report more fatigue while performing a fatiguing cognitive task and they adopt a more conservative response bias [31]. That is, MS patients require more “evidence” before releasing a response, and the relationship between effort and reward is different for persons with MS than for Controls. In MS the balance between effort and reward is persistently skewed because many aspects of cognition require increased effort. This results in persons with MS exhibiting a persistently skewed response bias. Interestingly, brain areas sensitive to fatigue (i.e., caudate) are also sensitive to response bias, which has implications for treatment (see below).

Can We treat fatigue?

If we don’t know what fatigue is, and we are not sure we are able to measure it, how can we treat it? In general, we can discuss two approaches: Pharmacological and Non-pharmacological Treatments.

Pharmacological approaches

Clinically, a pharmacological approach for symptoms of fatigue has been a mainstay of treatment. In general, three medications have been utilized the most in MS: amantadine, modafinil and methylphenidate. Despite its clinical utilization, most reviews show little to no support for any of these medications in the treatment of fatigue in MS (e.g., [37]). For instance, Modafinil is a wakefulness-promoting agent, approved for excessive sleepiness associated with narcolepsy, obstructive sleep apnea and shift-work disorder. Most studies lack support for modafinil as an effect treatment for fatigue in MS Moller et al. [25]. Despite this, a substantial number of MS patients are prescribed this treatment. Perhaps the seminal study was conducted by Nourbakhsh et al. [28] where in a randomized, double-blind, placebo-controlled crossover study, MS patients across centers received amantadine, modafinil, methylphenidate and a placebo (within group design). Results showed that all three medications were not superior to placebo but did result in more adverse events. They concluded that the results do not support the indiscriminate use of these medications to treat fatigue in MS. In fact, they more boldly concluded that: “Our results support the notion that most of the benefits that have been reported in the clinical use of medications for multiple sclerosis fatigue are attributable to the placebo effect.” The authors continue, “On the basis of our results, physicians should reduce the use of these medications for the treatment of multiple sclerosis fatigue”.

Similar lack of evidence exists in other populations such as stroke [24], Parkinson’s disease [15] and TBI (e.g., Canto et al. [6]). In fact, one study with TBI found *increased* fatigue as an adverse event of modafinil [21].

Non-pharmacological approaches

There is significant support for behavioral approaches to reduce fatigue in MS, namely: Cognitive Behavioral Therapy (CBT) and exercise. Harrison et al. [17] reviewed a host of behavioral interventions, in 6909 MS patients across 113 trials. CBT and balance exercise showed the best results with effect sizes in the moderate to large range. Similar approaches (aerobic exercise, relaxation, education or information) were less effective. Surprisingly, despite intuition, energy conservation has received little to no support in this or other studies (e.g., [5]). That is, recommending lifestyle alteration alone (e.g., exercise more, function only during periods of less fatigue or after rest) have little effect. Others have found support for CBT in MS (e.g., van den Akker et al. [35], Wendebourg et al. [36]) even in other populations (e.g., [37]) and exercise [9]. A recent review of 10 RCT’s found that mindfulness-based interventions were moderately effective for improving fatigue [34], and confirmed in a more recent review [29].

One novel approach to treating fatigue involves the reward system in the brain. It is well known that prolonged performance on cognitively demanding tasks often leads to mental or cognitive fatigue. That is, increasing time on task (ToT), typically results in higher levels of subjective fatigue. A growing body of literature suggests that the detrimental effects of ToT-induced fatigue can be reversed by increasing the task related motivational levels, such as providing rewards. Indeed, there is significant overlap between the reward system in the brain and the proposed fatigue network. Dobryakova et al. [13] showed that stimulation of the fronto-striatal network through monetary reward led to decreased fatigue in MS and healthy controls, which was associated with changes in brain activation in the ventral striatum and the ventromedial prefrontal cortex. Similar results were recently replicated by another group [11], and also observed in persons with TBI [14]. These studies show that motivational incentives (e.g., monetary reward) improve fatigue and are related to functional changes in the brain. Such data lead to a provocative hypothesis that central fatigue can be treated by using reward contingencies in MS patients experiencing fatigue. Such an approach could be individualized by determining what patients themselves find rewarding (e.g., what is it that they no longer engage in and would like to re-introduce) and employ behavioral techniques (cognitive behavior therapy) as a reward to reduce fatigue. This is potentially an intriguing and fruitful area of research.

## Conclusion and Suggestions

While fatigue is one of the most pervasive symptoms of MS affecting everyday life, its research and clinical application is mired by stagnation and a lack of innovation in its conceptualization and measurement. The answer to the question of can we measure fatigue and treat it; Yes, we must! However, we must gain a better understanding of the construct of fatigue. Clinically, we rely on subjective reports of fatigue, which is plagued by significant bias, resulting in instruments with poor content validity. What should we do?

Clinically, among other things, we need to acknowledge our limited understanding of our measurement tools. Self-report tools include variables unrelated to fatigue such as perceptions of depression, pain, medication, deconditioning and personality to name a few. We also need to know what fatigue is NOT. Fatigue can and should be differentiated from depression, sleep and muscle weakness, and acknowledge that fatigue does not necessarily result in actual cognitive or behavioral decline and is affected by personal factors such as motivation and personality. The complexity of fatigue must be considered when life altering decisions are required, such as whether to continue employment. It’s time to no longer view fatigue as a singular construct.

Scientifically, new research using functional imaging points to a fatigue network in the brain. Such a network could be very useful in clinical trials to assess treatment effects which could be used as a primary outcome of various interventions rather than self-report alone. Reliance on the current conceptualization of fatiguability may need to be revisited as it requires an a-priori stipulation that performance must decline, when it often does not, especially in the cognitive domain. Perceived fatigue and objective fatigability do not evaluate the same underlying concept.

Indeed fatigue has a significant impact on every day activities. However, it is our job as clinicians and researchers to truly understand this impact. That’s the challenge for the future.

## Data Availability

Not applicable. Not applicable.
