# Correction to: Fatigue in multiple sclerosis: can we measure it and can we treat it?

**DOI:** 10.1007/s00415-024-12630-8

**Published:** 2024-09-06

**Authors:** John DeLuca

**Affiliations:** 1 Kessler Foundation, 1199 Pleasant Valley Way, West Orange, NJ 07052 USA; 2 Department of Physical Medicine and Rehabilitation, New Jersey Medical School, Rutgers University, Newark, NJ USA

**Correction to: Journal of Neurology** 10.1007/s00415-024-12524-9

In the original version of this article, the caption of Fig. 1 was incorrect. Figure 1 caption should have read

**Fig. 1** Cortico-striato-thalamocortical (CSTC) loop underlying fatigue. Figure originally published in: Jameen ARM, Karen Ribbons, Jeannette Lechner-Scott, Saadallah Ramadan, Evaluation of MS related central fatigue using MR neuroimaging methods: Scoping review, Journal of the Neurological Sciences, Volume 400, 2019, Pages 52–71, 10.1016/j.jns.2019.03.007. Reused with permission from copyright holder. Crown Copyright © 2019 Published by Elsevier B.V. All rights reserved

